# Editorial: Methods and applications in vascular physiology: 2021

**DOI:** 10.3389/fphys.2022.1078988

**Published:** 2022-11-08

**Authors:** Julien V. Brugniaux, Rosalia Rodriguez-Rodriguez, Ali Dabiri, Kıvılcım Kılıç, Calum Wilson, Alexey Goltsov, Antonio Colantuoni

**Affiliations:** ^1^ HP2 laboratory (INSERM 1300), Université Grenoble Alpes, Saint Martin d’Hères, France; ^2^ Basic Sciences Department, Faculty of Medicine and Health Sciences, Universitat Internacional de Catalunya, Sant Cugat del Valles, Spain; ^3^ California Medical Innovations Institute, San Diego, CA, United States; ^4^ Biomedical Engineering Department, Boston University, Boston, MA, United States; ^5^ Strathclyde Institute of Pharmacy and Biomedical Sciences, University of Strathclyde, Glasgow, United Kingdom; ^6^ Institute for Artificial Intelligence, Russian Technological University (MIREA), Moscow, Russia; ^7^ University of Naples Federico II, Naples, Italy

**Keywords:** vascular physiology, methods, protocols, vascular disease, diagnosis, drug intervention

This Research Topic is a part of the Methods and Applications in Physiology series and aims at highlighting the latest experimental and computational methods in vascular physiology and their pharmacological, clinical and healthcare applications. The Research Topic includes methodological articles, describing either novel technical approaches or new applications of the conventional methods for measurement of vascular functions and parameters in health and pathological conditions. Since the vascular system is the main diagnostic and therapeutic target in much pathology, the most selected works directly relate to the clinical research and translation of the research methods to clinical practice. We hope that detailed discussion of methods and their applications in the separate Research Topic will be fruitful in relation to meeting one of the key challenges in experimental physiology, i.e., the development of reliable and rigorous methods, which ensure reproducibility of scientific results as well as unbiased and robust data analysis. Below, we briefly reviewed articles included in this Research Topic and presented main findings of the contributors.

Coagulation relies on the complex interaction of procoagulation and anticoagulation factors, as well as the fibrinolytic system. The normal clotting process goes through four phases among which, primary hemostasis and secondary hemostasis relate to clot formation. During the former, an unstable platelets plug forms at the site of injury. Indeed, within seconds to minutes after the injury, the blood vessels vasoconstriction and attract circulating platelets, which are activated, aggregate to one another and adhere to the subendothelium, eventually forming the platelet plug. During the first step of this process, the circulating platelets need to deposit to the exposed injured surface. The article from Wang et al. published herein, explores a novel mechanism that could regulate the deposition of platelets on the surface of the blood vessels. Due to the presence of sialic acid, the surface of platelets has an electronegatively charged and it has, therefore, been postulated by the authors that imposing an electric field (EF) could stimulate electrotaxis, i.e., the migration of the living cell towards cathode or anode. In this particular case, platelet deposition was reduced when the cathode of the EF was placed at the injured site, while it was increased should the anode be placed at the site. Most importantly, the authors found that to ensure only (or mostly) platelets would be deposited, the current needed to be less than 20 mV. Taken together, the present results suggest that manipulating the electric signal is an advantageous method to regulate the deposition of platelet onto the vessel wall.

Rajanathan et al. devoted their Methods paper to the development of an *in vivo* murine model for integrated assessment of the cardiovascular system in a pharmacological study of drug intervention. The *in vivo* model was used for the development of the protocol to investigate cardiac and vascular responses to the α_1_-agonist phenylephrine. Assessment of multiple intrinsic cardiac and vascular parameters allowed authors to validate the experimental protocol and distinguish phenylephrine-induced subsequent changes in peripheral vascular and cardiac systems. The developed method may contribute to the further progress in the integrated investigation of cardiovascular system responses to pharmacological intervention and testing new therapeutic targets.

Gu et al. described a developed protocol for a randomized clinical trial to study effectiveness of a mobile stroke unit (MSU) to manage acute stroke. This work focused on the comparison of clinical and economic outcomes of the innovative MSU based on 5G technology with a conventional emergency medical system. Authors see the advantages of the 5G-based MSU in combination of integrating neurological symptom examination, CT diagnosis, intravenous thrombolytic therapy, and remote consultation with the stroke centre through telemedicine system during patient transportation. Effective reducing the door-to-needle time and ensuring treatment within the golden time of stroke management are expected as the results of 5G-based MSU introduction in hospitals. This work extended the clinical trials on therapy to the trials on the effectiveness of the whole clinical unit to prove its benefits.

Nartsissov developed a computational method in vascular physiology to investigate dynamics of glucose supply to brain tissue. Author constructed a multi-scale computational model of glucose diffusion, transport and consumption in a neurovascular unit. In model construction, a multiphysical approach was utilised to combine different biophysical models to jointly describe hemodynamic in capillary, metabolite diffusion in heterogeneous tissue media and kinetics of biochemical reactions. The developed multi-scale computational approach may be extended to estimate distribution of different metabolites and drugs in brain tissue and used as a perspective computational tool in quantitative systems pharmacology (QSP) and PK/PD preclinical investigation.

The elegant review published by Masood et al. highlighted the importance of understanding lymphatic vessel regression in both physiological and pathological contexts, and explored the therapeutic potential of this knowledge. Considering the higher number of publications on the formation and regression of blood vessels compared to lymphatics, the authors proposed to extend insights from the fruitful field of blood vessels regression to shed light on the mechanisms underlying lymphatic vessel regression. Following these lessons, the potential mechanisms in lymphatic vessel regression may involve pro-survival and homeostatic signals, inflammation, anti-angiogenic switch and negative feedback, and apoptosis of regressing vessels. The authors also discussed the implications of newly identified roles of lymphatics in several pathologies, such as cardiovascular and neurodegenerative diseases, but importantly the potential avenues for therapeutic use of targeting lymphatic vessel regression. In particular, the use of appropriate models for the study of this process (e.g., mouse cornea, tail, bone or lung) has revealed the promising anti-lymphangiogenic effect of several drugs in pre-clinical and clinical studies in several pathologies such as lymphedema or Inflammatory Bowel Disease, but also in the prevention of tumour metastasis.

Palombo et al. have studied in ten young healthy volunteers, before and after a 5-week head-down tilt bed rest, the arterial pressure waveforms, which reflect the interaction between the heart and the arterial system with potentially relevant information about circulation conditions. According to the commonly accepted “wave transmission model,” the net blood pressure waveform results from the superposition of discrete forward and backward pressure waves, with the forward wave in systole determined mainly by the left ventricular ejection function and the backward by the wave reflection from the periphery, the timing and amplitude of which depend on arterial stiffness. However, this approach obscures the “windkessel function” (WF) of the elastic arteries. A “reservoir-excess pressure” (REP) model has been proposed, which interprets the arterial blood pressure waveform as a composite of a volume-related “reservoir” pressure and a wave-related “excess” pressure. The results in young people confirmed the hypothesis that REP analysis complements the wave-model adding a volume component related to the aorta WF, which may have variable impact and relevance according to age and health/disease status of the subjects.

Van der Laan et al. have improved their analysis method to automatically characterize vascular smooth muscle cells (VSMC) orientation and transmural distribution in murine carotid arteries under well-controlled biomechanical conditions. In the present study, they added a “nucleus splitting” procedure to split coinciding nuclei to increase the accuracy of their method. They tested this analysis technique in a mouse model of VSMC apoptosis. Carotid arteries from SM22a-hDTR Apoe−/− and control Apoe 37 −/− mice were dissected, excised, mounted in a biaxial biomechanical tester. Nuclei and elastin fibres were stained and imaged using 3D two-photon laser scanning microscopy. Nuclei were segmented from images and coincident nuclei were split. The nucleus splitting procedure increased the accuracy of the methods, in comparison with manual nucleus count. This improvement of the method may contribute to accurately determine the VSMC in the investigated vessels.

In a comparative study, Hersant et al. have found that in patients with suspected thoracic outlet syndrome (TOS) during the Candlestick-Prayer (Ca+Pra) maneuver, forearm pulse plethysmography, with red (R) or infrared (IR) light wavelength, was more accurate than fingertip pulse pletismography. They tested the effect of probe position (fingertip *versus* forearm) and compared the pattern classifications to the results of ultrasound. They recruited 20 patients for a Ca+Pra maneuver with simultaneous fingertip and forearm pulse pletismography (V-PPG) recording. V-PPG suggested the presence of venous outflow impairment in 27 and 20 limbs with forearm V-PPG-IR and forearm V-PPG-R, respectively. Fingertip V-PPG-R provided no patterns suggesting outflow impairment. The authors pointed out that probe position is essential if aiming to perform upper-limb V-PPG during the Ca+Pra maneuver in patients with suspected TOS. Moreover, V-PPG during the Ca+Pra maneuver is of low cost, easy and provides reliable and objective evidence of forearm swelling.

In a well-planned study, Zócalo and Bia have utilized ultrasound-derived blood flow velocity (BFV) levels (i.e., peak systolic velocity, PSV), intra-beat indexes (i.e., resistive) and inter-segment ratios (i.e., internal/common carotid artery (ICA/CCA) PSV ratio) to describe cardiovascular physiology and health status (i.e., disease severity evaluation and/or risk stratification). They pointed out that fixed cut-off values (disregard of age or sex) have been proposed to define “significant” vascular disease from BFV-derived data. The authors highlighted that the use of single fixed cut-off values has limitations. They suggested that an accurate use of BFV-derived parameters requires physiological age-related profiles and the expected values for a specific subject. They evaluated the difference between left and right data and calculated mean, standard deviation and age-related profiles for BFV levels in 3,619 patients. It is of interest that left and right body-side derived data were not always equivalent. Sex-specific reference interval (RI) was dependent on the parameter and/or age considered. RIs was defined for each studied artery: common carotid, internal carotid, external carotid, vertebral, femoral, and brachial arteries. They compared percentile curves with recommended fixed cut-off points. They reported equations for sex, body-side and age-specific BFV physiological profiles obtained in the investigated children, adolescents and adults, useful to determine the expected values and potential data-deviations for each person.

Taken together, the aforementioned articles showed a wide spectrum of the new experimental methods for measurement and analysis of multimodal data in vascular physiology. Looking at the successful issue of this Research Topic in 2021, we have launched the new Research Topic of Methods and Applications In Vascular Physiology: 2022 and welcome physiological experts to contribute and discuss the latest experimental, computational, statistical, and clinical methods in vascular physiology.

